# Multiple behavioral risk factors for non-communicable diseases among the adolescent population in Brazil: the analysis derived from the Brazilian national survey of school health 2019

**DOI:** 10.1186/s12887-024-04601-9

**Published:** 2024-02-15

**Authors:** Alanna Gomes da Silva, Juliana Bottoni Souza, Crizian Saar Gomes, Thales Philipe Rodrigues da Silva, Ana Carolina Micheletti Gomide Nogueira de Sá, Deborah Carvalho Malta

**Affiliations:** 1 Graduate Program in Nursing, Department of Maternal-Infant and Public Health Nursing, Federal University of Minas Gerais (UFMG), Avenida Professor Alfredo Balena, 190, Bairro Santa Efigênia, Belo Horizonte, 30130-100 Minas Gerais Brazil; 2 Faculty of Medicine, Graduate Program in Public Health, Federal University of Minas Gerais (UFMG), Belo Horizonte (MG), Brazil; 3 Federal University of São Paulo, Paulista School of Nursing, Rua Napoleão de Barros,754, Bairro Vila Clementino, São Paulo, 04023-062 Brazil; 4 Federal University of Minas Gerais (UFMG), School of Nursing, Department of Maternal-Infant and Public Health Nursing, Belo Horizonte (MG), Brazil; 5 School of Nursing, Graduate Program of the School of Nursing, Department of Maternal-Infant and Public Health Nursing, Federal University of Minas Gerais, Belo Horizonte (MG), Brazil

**Keywords:** Non-communicable diseases, Adolescent health, Risk factors, Brazil

## Abstract

**Background:**

Health risk behaviors often emerge or intensify during adolescence and tend to co-occur, exposing individuals to an even greater risk for the development of non-communicable diseases (NCDs). The likelihood of exhibiting multiple health risk factors also increases throughout life and is associated with sociodemographic characteristics contributing to their escalation and severity. In this context, the objective of this study was to analyze the association between sociodemographic characteristics and multiple behavioral risk factors for non-communicable diseases among the adolescent population in Brazil.

**Methods:**

This cross-sectional study utilized data from the Brazilian National Survey of School Health 2019. The sample comprised 121,580 adolescents aged 13 to 17. The analyzed variables included irregular intake of fruits and vegetables, regular consumption of soft drinks and treats, insufficient physical activity, sedentary lifestyle, cigarette smoking, and alcohol consumption. To analyze MBRFs, a classification ranging from zero to five was created, and associations were estimated using Odds Ratio (OR) with the respective 95% confidence interval (CI). The Backward method was employed for the multivariate regression model, utilizing ordinal logistic regression.

**Results:**

Adolescents without behavioral risk factors for NCDs constituted only 3.9% (95% CI 3.7–4.1). The most prevalent categories were two and three MBRFs, accounting for 28.3% (95% CI: 27.7–28.8) and 27.0% (95% CI: 26.5–27.5), respectively. Adolescents aged 16 and 17 (ORadj: 1.39; 95% CI: 1.32–1.48), residing in the Brazilian Southeast (ORadj: 1.66; 95% CI: 1.52–1.81), and those reporting poor or very poor self-rated health (ORadj: 2.05; 95% CI: 1.87–2.25) were more likely to exhibit multiple behavioral risk factors. Conversely, male adolescents (adjusted OR: 0.65; 95% CI: 0.62–0.69), those of mixed race (adjusted OR: 0.92; 95% CI: 0.87–0.97), and residents of rural areas (adjusted OR: 0.76; 95% CI: 0.70–0.84) were less likely to manifest MBRFs for NCDs.

**Conclusion:**

The majority of adolescents displayed MBRFs for NCDs, positively associated with age, region, and perceived health status. This underscores the necessity for healthcare promotional interventions throughout the life cycle, as these behaviors may persist into adulthood.

**Supplementary Information:**

The online version contains supplementary material available at 10.1186/s12887-024-04601-9.

## Introduction

Non-communicable diseases (NCDs) represent a significant health challenge in Brazil, mirroring global concerns, accounting for 75% of overall mortality (equivalent to 1,026,000 deaths) and 15% of premature deaths [1]. NCDs contribute to an increase in social inequalities, disability, hospitalization, and a reduction in quality of life and productivity [2].

NCDs have a multifactorial etiology, with modifiable behavioral risk factors prominently contributing to their causation, such as smoking, alcohol abuse, physical inactivity, and unhealthy diet [3, 4], along with social determinants like income, education, and environment, which contribute to the escalation and severity of NCDs, their risk factors, and morbidity and mortality [5–7].

Health behavior risks that predispose to poor health outcomes typically emerge during adolescence, a period marked by dynamic and complex physical, emotional, and social maturation, exposing individuals to new experiences [8]. Behavioral risk factors tend to co-occur and are interrelated, amplifying the risk of developing various diseases, including NCDs, due to their synergistic relationship [9]. There is also an increased likelihood of multiple hazardous health behaviors persisting throughout life [10].

Historically, numerous assessments have focused on promoting various healthy activities [11]. Studies in Brazil have explored the clustering of risk factors for NCDs among adolescents, revealing the simultaneous occurrence of various risk factors in their lives [12–16].

Understanding multiple behavioral risk factors (MBRFs) as predictors for NCDs carries significant implications for public health, enabling interventions based on comprehensive behavior modifications that are both more effective and economical [17–19]. Moreover, the unequal distribution of NCDs imposes public actions and policies aimed at preventing and reducing risk factors, improving access to healthcare, organizing surveillance and monitoring, and reducing social inequalities. It is crucial to comprehend the local reality to promote actions, plan services, and adapt policies to minimize the impact of these diseases and risk factors among adolescents, especially those less socially and economically privileged [7, 20]. Adolescence is a pivotal period for health promotion and the establishment of healthy habits.

This study aims to analyze the association between sociodemographic characteristics and multiple behavioral risk factors for non-communicable diseases among the adolescent population in Brazil.

## Methods

### Study design

This research is a cross-sectional study, utilizing data derived from the Brazilian National Survey of School Health (Pesquisa Nacional de Saúde do Escolar - PeNSE), carried out between April 9 and September 30, 2019.

### Setting

The PeNSE (Pesquisa Nacional de Saúde do Escolar) is a survey of adolescent pupils conducted by the Brazilian Institute of Geography and Statistics (IBGE), in collaboration with the Brazilian Ministry of Health and with the support of the Ministry of Education. This survey constitutes an integral part of the Surveillance of Risk and Protective Factors for Non-Communicable Diseases (NCDs) in Brazil. It stands out as the inaugural national survey that comprehensively addresses diverse aspects of adolescents’ lives, encompassing habits, care, as well as risk and protective factors influencing their health [21].

The survey’s sampling plan employed a two-stage cluster approach, with schools representing the initial stage of selection and the classes of enrolled students constituting the second stage. The student sample was derived from the selected classes. The sample size was determined to estimate population parameters for students aged 13 to 17, enrolled and attending both public and private schools, across various geographical levels, including the entirety of Brazil, the five major regions, Federative Units, capital cities, and the Brazilian Federal District [21].

The PeNSE questionnaire is self-administered and has specific filling guidelines [21].

### Participants

The survey included adolescent pupils aged 13 to 17, who were enrolled and regularly attending the 7th to 9th grades of Elementary School and the 1st to 3rd grades of High School. This encompassed Secondary Education with Technical Specialization and Teacher Training courses, across all shifts in both public and private schools throughout Brazil [21]. In 2019, the study collected data from a substantial number of institutions, comprising 4,242 schools and 6,612 classes. The participant pool involved 189,857 enrolled students, with 183,264 students in regular attendance. A total of 159,245 questionnaires were deemed valid, and 125,123 questionnaires were subjected to analysis.

Only adolescents who provided responses to all the questions were included in the analysis, and with missing answers were excluded. The final sample for this study comprised 121,580 adolescents.

### Variables

The following are the variables related to behavioral risk factors for NCDs, along with their respective questions and descriptions, as per the PeNSE:

1. Irregular intake of fruit and vegetables: Derived from two questions:

- “In the last 7 days, how many days did you eat at least one type of vegetable other than potatoes or cassava?”
- “In the last 7 days, how many days did you eat fresh fruit or fruit salad?”

Intake of fruit and vegetables on fewer than five days was considered irregular.

2) Regular consumption of soft drinks: Obtained from the question:

- “In the last 7 days, on how many days did you drink soft drinks?”

Consumption of soft drinks on five days or more was considered regular.

3) Regular consumption of treats: Obtained from the question:

- “In the last 7 days, on how many days did you eat sweet treats such as candies, confectionery, chocolates, chewing gums, bonbons, lollipops, and others?”

Consumption of treats on five days or more was considered regular.

4) Insufficient physical activity: Derived from the time spent in minutes per week on physical activity in three domains: traveling between home and school, physical education classes at school, and out-of-school physical activities. Obtained from four questions:

- “In the last 7 days, on how many days did you walk or bicycle to school?”
- “In the last 7 days, on how many days did you walk or bicycle back from school?”
- “In the last 7 days, on how many days did you take physical education classes at school?”
- “In the last 7 days, excluding physical education classes at school, on how many days did you take part in any physical activity?”

Those who did not engage in at least 300 min of physical activity per week were considered insufficiently active.

5) Sedentary lifestyle: Obtained from the question:

- “How many hours a day do you usually sit, watching television, playing video games, using a computer, mobile phone, tablet, or doing other activities while sitting down?”

Sedentary lifestyle was defined as sitting for three hours or more.

6) Current cigarette consumption: Obtained from the question:

- “In the last 30 days, on how many days did you smoke cigarettes?”

Current consumption was defined as those who had smoked on one or more days in the 30 days prior to the survey.

7) Current consumption of alcoholic beverages: Obtained from the question:

- “In the last 30 days, on how many days have you had at least one glass or one dose of alcoholic beverage?”

Current consumption of alcoholic beverages was defined as those who had consumed alcoholic beverages on at least one day in the 30 days prior to the survey.

The MBRFs for NCDs were obtained by summing the variables described previously: Irregular intake of fruit and vegetables; Regular consumption of soft drinks; Regular consumption of treats; Insufficient physical activity; Sedentary lifestyle; Current cigarette consumption and Current consumption of alcoholic beverages.

The assessment of MBRFs for NCDs involved utilizing a score ranging from zero to five, categorized as follows: 0 = no exposure; 1 = exposure to one factor; 2 = exposure to two factors; 3 = exposure to three factors; 4 = exposure to four factors; 5 = exposure to five, six, or seven risk factors.

The independent variables considered in the study encompassed self-declared gender (male and female), age group (13 to 15 years; 16 and 17 years), self-declared skin color (white, black, mixed race, and other - including yellow and native peoples), region (North, Northeast, Southeast, South, and Midwest), school administration (private and public), place of residence (urban and rural), and self-perception of health (very good or good, average, and bad or very bad).

### Statistical methods

Prevalence and their respective 95% confidence intervals (95% CI) for isolated and MBRFs were estimated for the overall adolescent population and stratified by independent variables.

Ordinal logistic regression was used to assess associations, with the Odds Ratio (OR) and respective 95% CI estimating the magnitude of these associations. The Backward method was employed for the construction of the multivariate regression model, where variables related to a level of statistical significance lower than 20% in the bivariate analysis were included and subsequently removed one by one.

All analyses were conducted using Stata, version 14.2, with the survey module considering post-stratification weights. Due to PeNSE’s complex sampling design and sample losses, post-stratification weights were applied to all analyses.

### Ethical considerations

PeNSE was approved by the Brazilian Ministry of Health’s National Research Ethics Committee for Human Beings, and informed consent was obtained from all participants and their legal guardian, opinion No. 3.249.268, dated 08 April 2019.

## Results

Among the adolescents participating in the survey, 50.7% (95% CI 49.9–51.4) identified as female, with the majority falling within the age range of 13 to 15 (64.7%, 95% CI 63.2-66.01). Additionally, a significant proportion had mixed skin color (43.6%, 95% CI 42.8–44.3), resided in the Southeast region (38.8%, 95% CI 37.7–39.9), attended public schools (85.5%, 95% CI 85.1–85.9), and self-perceived their health as very good or good (69.6%, 95% CI 68.9–70.2) (Table 1).

**Table 1:** Characteristics of adolescents participating in the national survey of school health. Brazil, 2019

Variables	%	95%CI
***Gender***		
Female	50.69	49.97–51.41
Male	49.31	48.59–50.03
***Age***		
13 a 15	64.70	63.22–66.14
16 e 17	35.30	33.86–36.78
***Race***		
White	35.99	35.18–36.81
Black	13.58	13.06–14.13
Mixed	43.56	42.82–44.3
Others	6.87	6.539–7.212
***Region***		
North	10.83	10.4-11.27
Northeast	28.35	27.61–29.11
Southeast	38.79	37.68–39.92
South	13.75	13.2-14.31
Midwest	8.28	7.97–8.592
***Administrative Unit***		
Private	14.46	14.09–14.84
Public	85.54	85.16–85.91
***Place of residence***		
Urban	92.42	90.96–93.67
Rural	7.58	6.334–9.044
***Self-assessment of health***		
Very good/good	69.55	68.94–70.15
Average	25.17	24.62–25.74
Bad/very bad	5.28	5.032–5.536

The most prevalent behavioral risk factor among adolescents was insufficient physical activity (71.5%, 95% CI 70.7–72.2), followed by irregular intake of fruit and vegetables (58.4%, 95% CI 57.7–59.0), sedentary lifestyle (54.1%, 95% CI 53.2–54.9), regular consumption of treats (32.9%, 95% CI 32.2–33.5), consumption of alcoholic beverages (28.1%, 95% CI 27.4–28.8), regular consumption of soft drinks (17.2%, 95% CI 16.7–17.8), and smoking cigarettes (6.8%, 95% CI 6.3–7.3) (Supplementary material).

Only 3.9% of adolescents had no behavioral risk factors for NCDs (95% CI 3.7–4.1). The most prevalent scenarios involved two or three simultaneous factors, accounting for 28.3% (95% CI: 27.7–28.8) and 27.0% (95% CI: 26.5–27.5), respectively.

Among adolescents who did not present any behavioral risk factors, 68.3% were male; 72.8% were 16 and 17 years old, 44.3% had mixed skin color; 36.6% lived in the Southeast region; 83.8% were public school students; 91.5% lived in urban areas and 84.4% self-perceiving their health status as very good or good. Among adolescents who presented five or more risk factors, 63.6% were women; 54.7% were 13 to 15 years old; 40.2% with white skin color; 48.2% lived in the Southeast region; 85.3% studied in public schools; 96.5% lived in urban areas and 55.1% self-perceiving their health status as very good or good (Table 2).

**Table 2:** Multiple behavioural risk factor for NCDs in adolescents, according to independent variables. National survey of school health. Brazil, 2019

	Quantity of behavioural risk factor for NCDs					
	0	1	2	3	4	5
	% (95%CI)	% (95%CI)	% (95%CI)	% (95%CI)	% (95%CI)	% (95%CI)
**TOTAL**	3.9 (3.7–4.1)	14.8 (14.3–15.3)	28.3 (27.7–28.8)	27.0 (26.5–27.5)	16.4 (16.2–17.1)	9.4 (9.0-9.9)
Gender*						
Female	31.7 (28.9–34.6)	42.0 (40.3–43.7)	48.3 (47.1–49.4)	52.2 (51.1–53.3)	57.6 (56.0-59.1)	63.6 (61.6–65.4)
Male	68.3 (65.4–71.1)	58.0 (56.3–59.6)	51.7 (50.5–52.8)	47.8 (46.7–48.9)	42.4 (40.8–43.9)	36.4 (34.5–38.3)
Age*						
13 a 15	72.8 (70.1–75.4)	71.0 (68.9–72.9)	68.1 (66.5–69.6)	62.9 (61.1–64.5)	61.0 (58.9–63.0)	54.7 (51.1–58.1)
16 e 17	27.2 (24.6–29.9)	29.0 (27.0-31.1)	31.9 (30.4–33.5)	37.1 (35.4–38.8)	39.0 (36.9–41.1)	45.3 (41.8–48.8)
Race*						
White	34.9 (32.1–37.7)	34.6 (33.0-36.2)	34.7 (33.4–35.9)	36.4 (35.2–37.5)	37.0 (35.6–38.3)	40.2 (38.0-42.5)
Black	12.9 (10.9–15.1)	12.7 (11.6–13.)	13.5 (12.5–14.4)	13.4 (12.6–14.3)	14.2 (13.2–15.1)	14.3 (12.7–15.9)
Mixed	44.3 (41.39–47.27)	46.0 (44.3-47.73)	44.7 (43.3–45.9)	43.9 (42.7–45.0)	41.9 (40.5–43.3)	39.2 (37.4–41.0)
Others	7.9 (6.367–9.682)	6.7 (5.9–7.5)	7.1 (6.5–7.7)	6.3 (5.7–6.8)	6.9 (6.1–7.6)	6.3(5.2–7.36)
Region*						
North	13.7 (12.0-15.3)	12.6 (11.5–13.7)	12.7 (11.9–13.3)	10.7 (10.0-11.4)	8.2 (7.6–8.8)	6.4 (5.7–7.3)
Northeast	28.1 (25.7–30.5)	30.7 (29.0-32.3)	32.2 (30.9–33.5)	28.1 (27.0-29.2)	25.2 (23.9–26.5)	19.3 (17.6–21.1)
Southeast	36.3 (33.1–39.5)	35.0 (32.7–37.3)	35.2 (33.6–36.8)	39.0 (37.5–40.4)	43.0 (41.2–44.8)	48.2 (45.6–50.7)
South	13.3 (11.4–15.4)	13.7 (12.5–14.9)	12.5 (11.7–13.3)	13.7 (12.9–14.5)	14.8 (13.7–15.8)	16.0 (14.6–17.5)
Midwest	8.6 (7.6–9.5)	8.0 (7.3–8.6)	7.4 (6.9–7.8)	8.5 (8.0-8.9)	8.8 (8.2–9.3)	9.9 (9.18–10.69)
Administrative Unit*						
Private	16.2 (14.7–17.6)	13.9 (13.1–14.6)	13.3 (12.7–13.9)	15.2 (14.6–15.7)	15.6 (14.8–16.3)	14.7 (13.7–15.7)
Public	83.8 (82.3–85.2)	86.1 (85.3–86.8)	86.7 (86.1–87.3)	84.8 (84.2–85.3)	84.4 (83.6–85.1)	85.3 (84.2–86.2)
Place of residence*						
Urban	91.5 (89.1–93.3)	90.6 (88.5–92.3)	90.2 (88.2–91.8)	92.7 (91.0–94.0)	95.3 (94.1–96.2)	96.5 (95.2–97.4)
Rural	8.5 (6.6–10.8)	9.4 (7.6–11.4)	9.8 (8.1–11.7)	7.3 (5.9–8.9)	4.7 (3.7–5.8)	3.5 (2.5–4.7)
Self-assessment of health*						
Very good/good	84.4 (82.3–86.2)	79.0 (77.7–80.2)	73.2 (72.0-74.3)	67.7 (66.6-68.9)	62.9 (61.7–64.1)	55.1 (52.8–57.2)
Average	13.9 (12.2–15.8)	17.8 (16.6–19.0)	22.8 (21.7–23.8)	26.8 (25.7–27.8)	30.2 (28.9–31.4)	35.5 (33.3–37.7)
Bad/very bad	1.7 (1.1–2.3)	3.2 (2.7–3.6)	4.0 (3.6–4.4)	5.5 (5.0–6.0)	6.9 (6.2–7.5)	9.4 (8.3–10.6)

* ***p***-value < 0.001

Table 3 presents the univariate analysis of MBRFs, revealing associations with gender, age (16–17 years), mixed race and others, Brazilian regions, school administrative unit, place of residence, and self-assessment of health. These findings underscore the impact of demographic and contextual factors on the prevalence of MBRFs among adolescents.

**Table 3:** Univariate ordinal logistic regression for the multiple behavioural risk factors for NCDs in adolescents. National survey of school health. Brazil, 2019

	%	95%CI	***p***-valor
***Gender***			
Female	*	*	
Male	0.61	0.58–0.64	0.000
***Age***			
13 a 15	*	*	
16 e 17	1.46	1.38–1.55	0.000
***Race***			
White	*	*	
Black	0.99	0.91–1.07	0.748
Mixed	0.87	0.83–0.91	0.000
Others	0.87	0.79–0.97	0.008
***Regions***			
North	*	*	
Northeast	1.15	1.07–1.23	0.000
Southeast	1.66	1.52–1.80	0.000
South	1.54	1.42–1.67	0.000
Midwest	1.57	1.46–1.69	0.000
***Administrative Unit***			
Private	*	*	
Public	0.93	0.88–0.98	0.004
***Place of residence***			
Urban	*	*	
Rural	0.61	0.56–0.67	0.000
***Self-assessment of health***			
Very good/good	*	*	
Average	1.76	1.66–1.87	0.000
Bad/very bad	2.28	2.09–2.50	0.000

* Reference group. %: percentage; 95%CI: 95% confidence interval

The final multivariate model, outlined in Table 4, reveals significant associations with MBRFs for NCDs among adolescents, indicating that adolescents aged 16 and 17 (ORadj: 1.39; 95% CI: 1.32–1.48), residing in all regions, particularly the Southeast (ORadj: 1.66; 95% CI: 1.52–1.81), and those with a self-perception of health as bad or very bad (ORadj: 2.05; 95% CI: 1.87–2.25) were more likely to exhibit MBRFs for NCDs. Conversely, male adolescents (ORadj: 0.65; 95% CI: 0.62–0.69), those of mixed race (ORadj: 0.92; 95% CI: 0.87–0.97), and residents of rural areas (ORadj: 0.76; 95% CI: 0.70–0.84) were less likely to have MBRFs for NCDs.

**Table 4:** Multivariate model for the multiple behavioural risk factors for NCDs in adolescents. National survey of school health. Brazil, 2019

	Odds Ratio		
	%	95%CI	***P***-Valor
***Gender***			
Female	*	*	
Male	0.65	0.62–0.69	0.000
***Age***			
13 a 15	*	*	
16 e 17	1.39	1.32–1.48	0.000
***Race***			
White	*	*	
Black	1.06	0.97–1.16	0.136
Mixed	0.92	0.87–0.97	0.005
Others	0.96	0.87–1.06	0.497
***Region***			
North	*	*	
Northeast	1.14	1.06–1.24	0.001
Southeast	1.66	1.52–1.81	0.000
South	1.54	1.40–1.69	0.000
Midwest	1.57	1.44–1.70	0.000
***Place of residence***			
Urban	*	*	
Rural	0.76	0.70–0.84	0.000
***Self-assessment of health***			
Very good/good	*	*	
Average	1.62	1.53–1.72	0.000
Bad/very bad	2.05	1.87–2.25	0.000

* Reference group. %: percentage; 95%CI: 95% confidence interval

## Discussion

This study aims to analyze the association between sociodemographic characteristics and multiple behavioral risk factors for non-communicable diseases among the adolescent population in Brazil. The study outcomes underscore a concerning reality, with only 3.9% of students exhibiting no behavioral risk factors for Non-Communicable Diseases and the majority displayed two or three MBRFs (Table 2). Notably, older adolescents, those residing in the Southeast, and those with a self-perception of health as bad or very bad were found to be more likely to harbor MBRFs for NCDs (Table 4).

Brazil faces ongoing challenges in promoting healthy behaviors among its adolescent population. A comparative analysis between the 2015 and 2019 editions of the National Survey of School Health (PeNSE) revealed a reduction in fruit and vegetable consumption, decreased physical activity, and an increase in episodes of drunkenness among adolescents. Furthermore, the prevalence of high consumption of ultra-processed foods persisted across all Brazilian states in 2019, ranging from 95.1% in Acre and Maranhão to 98.8% in São Paulo. The escalating use of alternative tobacco products, such as hookah and e-cigarettes, among young individuals is also a cause for concern and is observed nationwide. The diverse social, economic, and cultural landscape across Brazil’s regions plays a pivotal role in shaping health behaviors.

The Global School-based Student Health Survey revealed that 34.9% of adolescents globally had three or more simultaneous behavioral risk factors, with a prevalence of 56.2% in the Americas [24]. These findings raise concerns, as behaviors acquired during adolescence often persist into adulthood, contributing to heightened morbidity and mortality among young individuals. Consequently, understanding and addressing risk behaviors during adolescence are crucial for improving long-term health outcomes and reducing the burden of disease in adulthood [8]. Emphasizing a simultaneous approach to health promotion strategies is advocated, given their enhanced effectiveness, cost-efficiency, and greater impact on public health compared to interventions targeting isolated behaviors [25].

Notably, among adolescents aged 16 and 17, the likelihood of accumulating behavioral risk factors is higher (Table 4). Older adolescents are prone to exhibit habits associated with less healthy lifestyles, such as excessive screen time [26, 27], insufficient physical activity [28, 29], and alcohol and tobacco consumption [26]. This increased exposure to risk factors can be attributed to reduced social constraints imposed by parents or guardians during this stage, fostering greater independence in decision-making. Additionally, exposure to stressful situations, social pressures in late adolescence, and peer influence within their environment contribute to the heightened prevalence of risk behaviors [30, 31].

All five Brazilian regions had a positive and significant relation to the simultaneous presence of risk factors, especially the Southeast (Table 4). Urban areas also exhibited a higher simultaneous presence of risk factors among adolescents. The Southeast, with the highest urbanization rate and demographic density, reflects the challenges of accelerated and disorganized urban transition in the 20th century. This transition left a mark on the population, evident in unequal resource concentration, service disparities, and exacerbated environmental issues in the urban environment [32], potentially explaining the higher prevalence of this condition in the Southeast. The unplanned urban environment and city organization hinder the adoption of healthy habits, with some risk factors beyond individual choice, linked to unhealthy work areas or exposure to violence [33].

Examining living habits and residence of adolescents, a local study on food customs found that rural adolescents had a higher calorie intake from fresh or minimally processed foods [34]. Another research revealed that rural adolescents, compared to their urban counterparts, used fewer electronic devices, spent less time sedentary, and were less likely to be insufficiently active [35]. Urban environmental characteristics, such as security issues, high population density, increased access to the internet, computers, and electronic games, and more obesogenic environments, may contribute to decreased physical activity, increased sedentary behavior, unhealthy food intake, and alcohol consumption among adolescents [32, 36]. Therefore, the increase in behavioral risk factors is considered multifactorial, influenced by societal organization and urban lifestyles.

Perceived health status, a subjective measure reflecting individuals’ views of their health, serves as an essential indicator. Although it may not directly indicate actual health status, its relationship with other health and socio-demographic characteristics can reflect healthy habits or risk behaviors. Individuals with better socioeconomic conditions, healthy behaviors, and no comorbidities generally have a better self-perception of health. Conversely, those with NCDs, low levels of schooling, smokers, and inadequate fruit and vegetable consumption tend to rate their health worse [37]. Brazilian adolescents exhibited a similar pattern, linking poor self-rated health to health risk behaviors such as regular alcohol and soft drink consumption, experimentation with illicit drugs, and issues related to physical and emotional health [38]. This underscores the population’s ability to relate lifestyles to their well-being and health assessment.

The Covid-19 pandemic has negatively impacted lifestyles of adolescents, leading to increased consumption of frozen food, chocolates, sweets, extended screen time, and subsequently, reduced physical activity [39]. This can result in weight gain, loss of cardiorespiratory fitness, sleep issues, and adversely affect the growth, development, and social health of adolescents, potentially persisting throughout their lives [40–42]. This situation may have intensified during the pandemic, further increasing the likelihood of accumulating risk factors for NCDs. Continuously monitoring the health conditions of adolescents is crucial to inform and guide education and health interventions for this group, aiming to minimize the adverse effects brought about by the pandemic. Additionally, implementing public policies and new educational approaches that promote a healthy lifestyle among adolescents is essential, rather than simply reverting to pre-COVID-19 strategies [41].

Limitations of this study includes potential recall bias and the use of a self-administered questionnaire, which might lead to students misinterpreting questions and result in underestimates or overestimates of the studied indicators. However, it’s important to note that PeNSE is based on well-established international surveys like the Global School-Based Student Health Survey, Health Behaviour in School-Aged Children, and Youth Risk Behavior Surveillance System. These surveys have validated the questionnaire, demonstrating satisfactory results in reproducibility and validity analyses. Additionally, PeNSE focuses on students regularly enrolled and attending school in Brazil, excluding those without this educational link, potentially missing more vulnerable adolescents. Nevertheless, the survey includes schools in indigenous and remote areas, with an expanded sample in the 2019 edition, providing insights over major regions, states, and capital municipalities. Despite its limitations, PeNSE offers a valuable snapshot of the reality of school-attending adolescents aged 13 to 17.

## Conclusion

Over 80% of Brazilian adolescents presented multiple behavioral risk factors for non-communicable diseases, with increased likelihood observed among older adolescents, those residing in the Southeast, and those reporting poorer self-perception of health. This elevated prevalence underscores the imperative for health promotion initiatives spanning the entire life cycle. Furthermore, there is a pressing need for dynamic and proactive approaches that empower adolescents to take co-responsibility for their health. Concurrently, the implementation of intersectoral policies is crucial to adopt improved living and health conditions.

### Electronic supplementary material

Below is the link to the electronic supplementary material.

Supplementary Material 1

## Data Availability

The datasets generated and/or analysed during the current study are available in the [Brazilian Institute of Geography and Statistics (IBGE)] repository, [https://www.ibge.gov.br/estatisticas/sociais/saude/9134-pesquisa-nacional-de-saude-do-escolar.html?edicao=31442&t=resultados]

## Abbreviations

PeNSE: National Survey of School Health
NCDs: Non-Communicable Diseases
95%CI: 95% confidence intervals
ORb: Crude Odds Ratio
ORaj: Adjusted Odds Ratio
