# Supplementary material for: Multiple behavioral risk factors for non-communicable diseases among the adolescent population in Brazil: the analysis derived from the Brazilian national survey of school health 2019
Source: BMC Pediatr. 2024 Feb 15;24:122. doi: 10.1186/s12887-024-04601-9 (PMC10868108; doi:10.1186/s12887-024-04601-9)
Supplement: Supplementary file 1 — Supplementary Material 1 [file 12887_2024_4601_MOESM1_ESM.docx]

Supplementary material. Behavioral risk factors for NCDs in adolescentes, according to independent variables. National Survey of School Health. Brazil. 2019

|  | **Insufficient physical activity** | **Irregular intake of fruit and vegetables** | **Sedentary lifestyle** | **Regular consumption of treats** | **Current consumption of alcoholic beverages** | **Regular consumption of soft drinks** | **Current cigarette consumption** |
| --- | --- | --- | --- | --- | --- | --- | --- |
|  | **%(95%CI)** | **%(95%CI)** | **%(95%CI)** | **%(95%CI)** | **%(95%CI)** | **%(95%CI)** | **%(95%CI)** |
| ***TOTAL*** | 71.5(70.7-72.2) | 58.4(57.7-59) | 54.1(53.2-54.9) | 32.9(32.2-33.5) | 28.1(27.4-28.8) | 17.2(16.7-17.8) | 6.8(6.3-7.3) |
| ***Gender*** |  |  |  |  |  |  |  |
| Female | 81.7(80.9-82.5) | 59.3(58.4-60.2) | 55.3(54.3-56.2) | 38.1(37.2-38.9) | 30.1(29.2-31.1) | 16.3(15.7-17) | 6.5(5.8-7.3) |
| Male | 60.9(59.9-61.9) | 57.4(56.5-58.3) | 52.8(51.7-53.9) | 27.5(26.7-28.3) | 26(25.2-26.8) | 18.1(17.4-18.9) | 7.1(6.6-7.6) |
| ***Age*** |  |  |  |  |  |  |  |
| 13 a 15 | 71.0(70.2-71.8) | 56.8(56.1-57.6) | 53.7(52.7-54.8) | 32.8(32-33.7) | 22.2(21.5-22.9) | 17.1(16.4-17.8) | 5.0(4.5-5.7) |
| 16 e 17 | 72.3(70.9-73.7) | 61.2(60.1-62.2) | 54.6(53.4-55.8) | 32.9(32-33.8) | 38.9(37.6-40.3) | 17.4(16.5-18.3) | 10(9.3-10.8) |
| ***Race*** |  |  |  |  |  |  |  |
| White | 71.7(70.5-72.8) | 55.9(55-56.9) | 58.2(57.1-59.3) | 33.8(32.9-34.8) | 30(29.1-31) | 18.4(17.6-19.3) | 6.4(5.8-7.2) |
| Black | 68.7(67-70.3) | 61.5(60-62.9) | 52.3(50.4-54.3) | 34(32.4-35.6) | 30.5(28.8-32.2) | 18.3(16.9-19.8) | 8.3(7.4-9.3) |
| Mixed | 72.2(71.1-73.3) | 59.1(58.2-60) | 51.8(50.7-52.9) | 32(31-32.9) | 26.2(25.2-27.2) | 15.9(15.2-16.7) | 6.6(6.1-7.2) |
| Others | 71.6(69.3-73.8) | 60.6(58.4-62.7) | 50.6(48-53.2) | 32.1(30-34.2) | 25.4(23.3-27.6) | 17(15.3-18.9) | 6.6(5.6-7.8) |
| ***Region*** |  |  |  |  |  |  |  |
| North | 72(70.5-73.5) | 60.4(58.5-62.2) | 44.4(42.2-46.6) | 26.9(25.7-28.1) | 19.3(18.3-20.4) | 12.9(12-13.8) | 7.2(6.4-8.1) |
| Northeast | 75.5(74.5-76.5) | 63.4(62.4-64.3) | 47.4(46-48.9) | 28.2(26.9-29.4) | 22.1(21-23.2) | 12.3(11.6-13) | 4.7(4.3-5.2) |
| Southeast | 70.2(68.7-71.7) | 56.5(55.3-57.8) | 60.2(58.6-61.7) | 36.3(35-37.6) | 30.8(29.3-32.4) | 20.9(19.6-22.2) | 7.6(6.5-8.8) |
| South | 66.8(64.8-68.7) | 54.2(52.8-55.5) | 57(55.1-58.9) | 34.8(33.4-36.2) | 38.4(36.6-40.4) | 17.6(16.4-18.9) | 8(7.1-9) |
| Midwest | 70.4(69.1-71.8) | 54.2(52.8-55.6) | 55.8(54.4-57.1) | 37.5(36.3-38.7) | 30.3(29.1-31.6) | 21.7(20.8-22.7) | 7.7(7-8.5) |
| ***Administrative Unit*** | |  |  |  |  |  |  |
| Private | 71.3(70.4-72.2) | 51.8(51-52.6) | 63.6(62.6-64.5) | 35.9(34.9-37) | 27.7(26.7-28.7) | 17.4(16.7-18.1) | 4.4(4.1-4.8) |
| Public | 71.5(70.6-72.4) | 59.5(58.8-60.2) | 52.4(51.5-53.4) | 32.3(31.6-33.1) | 28.2(27.3-29) | 17.2(16.5-17.9) | 7.2(6.6-7.8) |
| ***Place of residence*** | |  |  |  |  |  |  |
| Urban | 70.9(70.1-71.7) | 58.1(57.4-58.7) | 55.9(55-56.8) | 33.6(32.9-34.3) | 28.8(28-29.6) | 17.7(17.1-18.3) | 6.8(6.3-7.3) |
| Rural | 78.4(76.3-80.4) | 62.1(59.4-64.8) | 31.7(28.7-34.9) | 23.7(22.1-25.5) | 19.2(17.1-21.6) | 10.9(9.6-12.4) | 7.2(5.8-8.7) |
| ***Self-assessment of health*** | |  |  |  |  |  |  |
| Very good/good | 69(68.2-69.8) | 55.4(54.7-56.2) | 51.3(50.3-52.2) | 31(30.3-31.8) | 25.6(24.8-26.3) | 16.6(15.9-17.4) | 5.8(5.4-6.3) |
| Average | 77.3(76.2-78.4) | 64.8(63.7-65.9) | 60.4(59.1-61.7) | 36.5(35.3-37.7) | 32.9(31.6-34.3) | 17.7(16.7-18.7) | 8.2(7.4-9.1) |
| Bad/very bad | 77.6(75.2-79.7) | 66.6(64.6-68.6) | 63.3(60.9-65.6) | 39.6(37.2-41.9) | 37.8(35.4-40.3) | 21.3(19.3-23.4) | 11.9(10.2-13.8) |
